# Pathogenesis and immune regulation of rheumatoid arthritis-associated interstitial lung disease: from basic research to clinical implications

**DOI:** 10.3389/fimmu.2026.1770348

**Published:** 2026-03-13

**Authors:** Yaxin Cheng, Yu Shan, Yixin Zheng, Fuyu Zhao, Yiming Shi, Chenyang Song, Yunshen Li, Jianan Zhao, Cen Chang, Yuguang Wang, Dongyi He

**Affiliations:** 1 Guanghua Hospital Affiliated to Shanghai University of Traditional Chinese Medicine, Shanghai, China; 2 Department of Respiratory, Beijing Hospital of Traditional Chinese Medicine, Capital Medical University, Beijing, China

**Keywords:** rheumatoid arthritis-associated interstitial lung disease, immune regulation, tertiary lymphoid organs, autoantibody, pulmonary fibrosis

## Abstract

Interstitial lung disease (ILD) is one of the most common extra-articular manifestations of rheumatoid arthritis (RA). Some patients with RA-ILD may develop progressive pulmonary fibrosis, leading to severe impairment of lung function and respiratory failure, which impacts quality of life and can even be life-threatening. This review identified genetic susceptibility, environmental factors, and immune dysregulation as key contributors to the etiology and pathogenesis of RA-ILD. We highlight that autoantibodies, adaptive immune abnormalities, and tertiary lymphoid organ formation significantly drive pulmonary inflammation and fibrosis, while pro-inflammatory cytokines and epithelial-mesenchymal transition (EMT) further contribute to lung tissue injury. Current treatment options, including glucocorticoids, immunosuppressants, and antifibrotic agents such as nintedanib and pirfenidone, are often limited by substantial side effects. Additionally, emerging therapies like JAK inhibitors, CAR-T cells, and the upcoming phosphodiesterase-4B inhibitor, nerandomilast, show promise, but no curative treatment exists to date. Future research could focus on multi-omics technologies and conducting multicenter clinical trials to establish therapeutic targets and advance precision medicine for RA-ILD.

## 1 Introduction

Rheumatoid arthritis-associated interstitial lung disease (RA-ILD) is a common extra-articular manifestation of RA. According to a long-term follow-up studies of RA cohorts, the cumulative risk of developing clinical ILD during the course of the disease has ranged from 5.0% to 7.7%, and a study using death records found that it could reach 10%. Patients with RA have also been found to have even higher estimates for subclinical radiographic findings consistent with ILD, ranging from 19% to 33% (1). And over time, the cumulative incidence of ILD in RA patients continues to rise (2). Some patients experience progressively worsening symptoms, including cough, shortness of breath, difficulty breathing, sleep disturbances, fatigue, anxiety, and depression, which severely impact their quality of life (3, 4).

ILD constitutes a major cause of mortality in RA patients, 35.9% of RA-ILD patients survive less than 5 years post-diagnosis, with a median survival of merely 7.8 years (5). A cohort study shows that mortality disparities are evident: RA-ILD patients have a 1-year and 10-year mortality rate of 13.9% and 60.1%, respectively, while non-ILD RA counterparts have a 3.8% and 34.5% mortality rate. The hazard ratio for mortality increases 2- to 10-fold in RA-ILD populations (6). These findings underscore ILD’s dual burden—significantly compromising quality of life while dramatically accelerating mortality. However, therapeutic options remain limited for RA-ILD, the primary drugs currently used in clinical practice, such as glucocorticoids and immunosuppressants, failed to achieve a curative effect.

Recent studies have highlighted the pivotal role of immune-inflammatory processes in driving the pathogenesis of RA-ILD, key contributors include pathogenic autoantibody production, dysregulated adaptive immunity and pro-fibrotic molecular pathways (7). Nevertheless, the exact interplay of these mechanisms remains incompletely elucidated. In this review, we focus on the etiological factors and immunological mechanisms underlying RA-ILD, as well as controversy over current medication and potential therapeutic targets.

## 2 Etiological factors of RA-ILD

### 2.1 Genetic susceptibility

**Figure 1:**
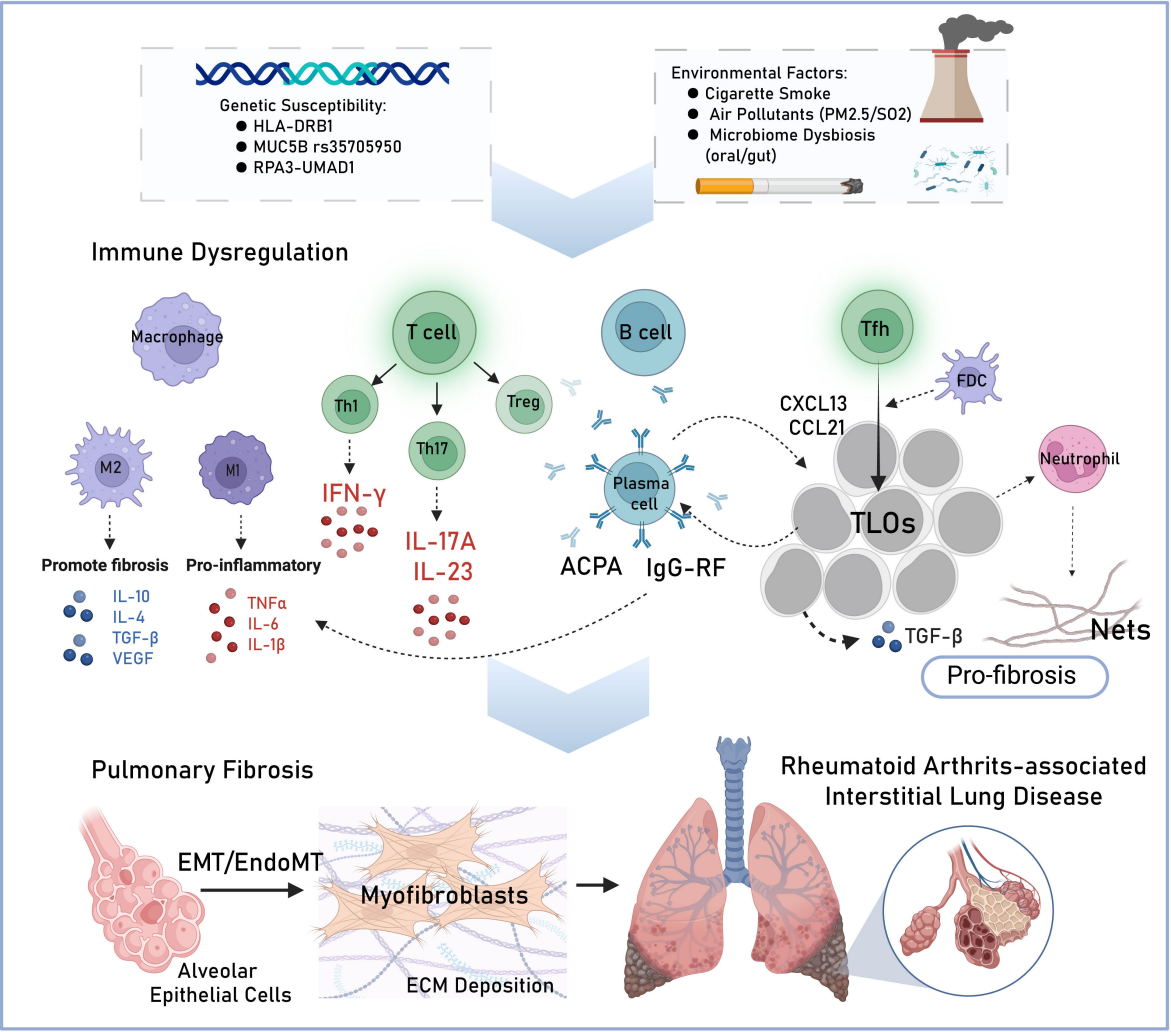
Schematic diagram of risk factors and proposed pathogenesis of rheumatoid arthritis-interstitial lung disease (RA-ILD). Genetic susceptibility and environmental factors (such as cigarette, PM2.5 etc.) are key contributors to the etiology of RA-ILD. In the pathogenesis, adaptive immune abnormalities drive a drive a sustained immune-inflammatory response, activating T cells, B cells, macrophages and neutrophil, which releasing pro-inflammatory and pro-fibrosis factors. The formation of tertiary lymphoid organs (TLOs) further amplifies pulmonary inflammation and fibrosis. Pro-inflammatory cytokines together with epithelial-mesenchymal transition (EMT) and endothelial-to-mesenchymal transition (EndoMT) contribute to lung tissue injury and excessive extracellular matrix (ECM) deposition. ACPA, anti-citrullinated protein antibodies; RF, rheumatoid factor; Tfh, follicular helper T cells; FDC, follicular dendritic cells; Nets, neutrophil extracellular traps.

Despite the precise molecular mechanisms underlying RA-ILD pathogenesis remain incompletely characterized, accumulating evidence supports a tripartite pathogenic framework involving genetic susceptibility, environmental triggers and dysregulated autoimmune responses (**Figure 1**). Genetic factors, mainly in the class II major histocompatibility complex (MHC) region, confer a risk of up to 50% for the development of RA (8). Specifically, the human leukocyte antigen (HLA)-DRB1 allele, a key regulator of antigen presentation and T-cell immune responses, exists a strong association with RA-ILD progression (9, 10).Emerging genetic studies have identified multiple susceptibility loci where single nucleotide polymorphisms (SNPs) significantly influence RA-ILD pathogenesis, including the promoter variant rs35705950 of mucoprotein 5B *(MUC5B)* (11), which leads to varying rates of RA-ILD in Northern European, American and Aisan populations (11–13). Genome-wide association studies have also identified rs6578890 in the PPFIA1-binding protein 2 (*PPFIBP2*) gene and *rs12702634* at *RPA3-UMAD1* as risk factors for developing RA-ILD in different regions (7, 14, 15). Most interestingly, the first whole exome sequencing study of RA-ILD patients revealed a significant prevalence of mutations in telomerase reverse transcriptase (*TERT)*, regulator of telomere-elongation helicase-1 (*RTEL1)*, polyadenylation-specific ribonuclease deadenylation nuclease (*PARN)*, and surfactant protein C (*SFTPC)*. Telomeres were shorter in RA-ILD patients with *TERT*, *RTEL1*, or *PARN* mutations compared to controls, highlighting shared genetic risk factors between RA-ILD and familial pulmonary fibrosis (16).

While genetic factors play a significant role in RA-ILD susceptibility, other aspects such as sex also influence disease outcomes. RA predominantly affects females, but male sex has been identified as an independent predictor significantly associated with RA-ILD, serving as a robust variable for predicting concurrent pulmonary manifestations (including ILD, pulmonary nodules, and bronchiectasis) in RA patients (17).

### 2.2 Environmental exposures

Smoking has been recognized as a major environmental risk factor for RA-ILD, with the risk increasing with cumulative cigarette exposure and persisting even after smoking cessation. Cigarette components, especially polycyclic aromatic hydrocarbons (PAHs), contribute to RA pathogenesis by triggering inflammatory pathways in synovial dendritic cells (18). Smokers, particularly those with a history of heavy smoking, have a higher risk of developing RA-ILD due to increased autoimmunity and airway inflammation (19). Existing studies also comprehensively demonstrated other specific environmental factors (including exposure to air pollutants such as PM2.5, PM10, SO2, and NO2), along with advanced age and male sex are associated with increased RA-ILD risk (20). Distinct microbial profiles in oral (21), pulmonary (22), and gut microbiomes (23) have been observed between RA and RA-ILD patients, leading researchers to hypothesize that microbe-induced antigen-antibody cross-reactions may trigger autoimmune activation and contribute to RA-ILD pathogenesis.

### 2.3 Autoimmune dysregulation

Autoantibodies, such as anti-citrullinated protein antibodies (ACPA) and rheumatoid factor (RF), are currently key indicators for the clinical diagnosis of RA (24). Previous study already reported that both positive RF (HRs = 1.15, 95%CI 0.75-1.77; ORs = 2.11, 95%CI 1.65-2.68) and positive ACPA (ORs = 2.11, 95%CI 1.65-2.68) were identified as risk factors for RA-ILD (25). These autoantibodies were even more closely associated with severe ILD (Forced vital capacity < 80%) (26). The elevated levels of these and other specific autoantibodies demonstrate disease-specific association with RA-ILD, highlighting their potential as clinically prognostic biomarkers (20, 27). Recently, a cross-sectional study identified several peripheral biomarker signatures significantly associated with RA‐ILD, including anti-malondialdehyde-acetaldehyde adduct (AMMA), ACPA and RF (28). In addition, positive antineutrophil cytoplasmic antibody (ANCA) as well exhibited significantly elevated inflammatory markers and higher disease activity scores compared to ANCA-negative counterparts, along with more severe pulmonary function impairment (29). Another study reported significantly higher levels of Exosome component 4 (EXOSC4) antibodies in the sera of RA-ILD patients, positively correlated with inflammatory markers, suggesting that EXOSC4 may serve as a potential autoantigen in RA-ILD (30). Collectively, these risk factors may drive disease progression by promoting pulmonary inflammation and fibrotic processes.

## 3 The pathogenesis of RA-ILD

Among all internal organs, the lungs are uniquely exposed to the external environment, making them susceptible to diseases triggered by alveolar epithelial injury or immune dysregulation from various factors. Most interstitial lung diseases are characterized by alternating phases of inflammation and fibrosis, So is RA-ILD (31). However, how autoantibodies and adaptive immunity promote the progress of lung inflammation and fibrosis is very complicated, which will be further clarified in this review.

### 3.1 Role of autoantibodies in RA - ILD

Citrullinated proteins, recognized as “non-self” by the immune system, trigger the production of ACPAs by plasma cells, the ACPA-positive RA patients are at a higher risk for extra-articular manifestations, including pulmonary involvement (32, 33). It may drive RA-ILD progression through multiple mechanisms (34). Firstly, citrullinated proteins bind to ACPA, forming immune complexes that trigger an inflammatory cascade involving cytokines like TNF-α and IL-1β (35). Subsequently, sustained inflammatory responses promote the infiltration of CD4^+^ T cells, B cells, neutrophils, and macrophages into the pulmonary interstitium. Through the secretion of cytokines and chemokines, creating a chronic inflammatory microenvironment (36). Inflammatory mediators, including TNF-α, promote fibroblast proliferation, extracellular matrix degradation. They also induce the release of pro-fibrotic factors (PDGF-β, TGF-β, VEGF), which drive fibroblast transformation into collagen-producing myofibroblasts via autocrine/paracrine signaling, leading to abnormal collagen buildup in lung tissue and accelerating RA-ILD progression (35).

RF is an autoantibodies with Fc fragment of denatured IgG as target antigen, plays a dual role in RA pathogenesis by affecting both joints and extra-articular tissues (37). Notably, RF demonstrates significant involvement in the development of ILD, with its serum titers showing strong correlation with radiographic progression of RA-ILD (38, 39). In terms of pathogenic mechanisms, RF forms immune complexes that deposit in lung tissue, activating the complement system and recruiting inflammatory cells such as neutrophils and macrophages. This leading to the release of pro-inflammatory cytokines and MMPs, resulting in alveolitis and interstitial lung damage. Antigen-antibody complexes are recognized by B cell surface receptors, promoting continuous antibody secretion and forming a positive feedback loop. Additionally, RF may cross-react with other autoantibodies (such as ACPAs antibodies) to activate fibroblasts and promote abnormal extracellular matrix deposition, driving pulmonary fibrosis. Thus, its dual role in inflammation and fibrosis in RA-ILD is central to the link between extra-articular autoimmunity and lung tissue damage (40). In addition, EXOSC4 antibodies also increased in RA-ILD as we mentioned before, researchers identified it EXOSC4 may exacerbate the fibrosis process by activating the Wnt/β-catenin signaling pathway, which in turn affects the proliferation and differentiation of Alveolar cells (30).

### 3.2 The role of adaptive immune responses in RA-ILD

Adaptive immune responses play a crucial role in the pathogenesis of RA-ILD, particularly the imbalance of lymphocyte subsets and the inflammatory responses they mediate, which may promote the pulmonary fibrotic process. For example, Sumida et al. (41) demonstrated that fibroblasts could be induced by Th1-mediated immune response in patients with nonspecific interstitial pneumonia. And Yaxiong Jin et al. (42) found an elevated Th17/Treg ratio was also independent risk factors for RA-ILD patients. Abnormal changes in the T cell receptor signaling pathway, especially the activation of pathways such as PI3K-Akt and MAPK, further augment the release of inflammatory cytokines and lung tissue damage (43). Furthermore, a recent single-cell study have uncovered more specialized T-cell subsets involved in RA-ILD. Notably, peripheral helper T cells (Tph), were found to be exclusively enriched in the lung tissue of RA-ILD patients compared to those with non-RA connective tissue disease (CTD)-ILD patients, highlighting a potentially unique T-cell-driven pathological mechanism in RA-ILD (44). Unlike T cell, the role of B cells in RA-ILD is mainly through the production of autoantibodies, and their dysregulated function under disease conditions leads to the persistence of inflammatory responses (45). Adaptive immune responses interact with innate immune cells in RA-ILD, such as macrophages and NK cells, play a crucial role in promoting disease progression. M1 macrophages secrete proinflammatory cytokines such as IL-6 and TNF-α, which exacerbate inflammation and fibrosis in the lungs, while M2 macrophages contribute to fibrosis by secreting profibrotic cytokines like TGF-β. The interaction between these macrophage subpopulations, along with the activation of the Th1/Th17 pathway, amplifies the cytokine storm and drives the development of fibrosis in the lung parenchyma (18).

### 3.3 Formation and significance of tertiary lymphoid organs in RA - ILD

Tertiary lymphoid tissues (TLOs), which also called “inducible bronchus associated lymphoid tissue (iBALT)”, are most well-developed and prevalent in those with pulmonary complications of RA (46). The formation of TLOs primarily depends on the aggregation of follicular helper T cells (Tfh), B cells, and follicular dendritic cells (FDCs), Tfh cells recruit B cells and T cells by secreting chemokines (e.g., CXCL13 and CCL21), thereby promoting TLOs formation (7, 46). TLOs play a critical role in the pathogenesis of RA-ILD. At first, TLOs serve as important sites for local production of autoantibodies. On one hand, Tfh cells within TLOs assist B cells in differentiating into plasma cells that produce autoantibodies, which exacerbates citrullination and oxidative stress in lung tissue. This also stimulates neutrophils to release neutrophil extracellular traps (NETs). On the other hand, the interactions between B cells and T cells within TLOs promote the secretion of pro-inflammatory cytokines, such as IL-17 and TNF-α, which further contribute to lung tissue damage (7). Furthermore, the presence of TLOs is closely linked to the progression of pulmonary fibrosis. Studies indicate that immune cells within TLOs release pro-fibrotic factors, such as TGF-β and NETs, inducing the fibroblast-to-myofibroblast transition (FMT), which promotes extracellular matrix deposition and ultimately leads to pulmonary fibrosis (47). Given the pivotal role of TLOs in RA-ILD, therapeutic strategies targeting TLOs may hold significant clinical potential. For instance, inhibiting CXCL13 or blocking the function of Tfh cells could reduce TLO formation, thus alleviating pulmonary inflammation and fibrosis.

### 3.4 Molecular mechanisms of pulmonary fibrosis in RA - ILD

The initiation step in the pathogenesis of RA-ILD involves risk factors such as smoking and environmental pollutants, which trigger damage to airway and alveolar epithelial cells in genetically susceptible individuals. Variations in gene loci such as MUC5B may hinder the repair process of alveoli, reducing their ability to shield and process pathogenic bacteria, thus increasing susceptibility to pulmonary fibrosis. Then leads to persistent epithelial cell damage and activation of immune cells (including neutrophils, dendritic cells, and macrophages), resulting in the excessive accumulation of extracellular matrix (ECM) components in lung tissue (48). For instance, a recent study by Aripova et al. (49) found that fibrinogen modified by citrulline and/or MAA can activate macrophages, leading to the secretion of platelet-derived growth factor and TGF-β, which in turn upregulates the gene expression of CD36, COL6A3, MMP-9, MMP-10, and MMP-12 in human lung fibroblasts (HLF). HLF cells produce more type I/VI collagen deposits, suggesting that the interaction between activated macrophages and human lung fibroblasts leads to significant ECM deposition and promotes the progression of pulmonary fibrosis (49).

The accumulation of a large number of myofibroblasts is responsible for exaggerated and uncontrolled production of ECM during the development and progression of RA-ILD. Transition of epithelial cells into mesenchymal cells (epithelial-mesenchymal transition, EMT) plays a critical role in the development and progression of various lung diseases, contributing to the accumulation of myofibroblasts and subsequent ECM deposition. Therefore, EMT is considered one of the key processes in the pathogenesis of RA-ILD (20). Cytokines, including IL-17, IL-17A, IL-23, and growth factors such as TGF-β, can amplify tissue damage and promote pulmonary mesenchymal expansion through the EMT process (50, 51). Recently, endothelial-to-mesenchymal transition (EndoMT), a newly recognized type of cellular transdifferentiation, has emerged as another possible source of tissue myofibroblasts. The frequency of endothelial progenitor cells (EPC) is increased in RA-ILD and idiopathic pulmonary fibrosis (IPF) patients; it may represent a reparative compensatory mechanism (52). Therefore, genetic susceptibility, immune dysregulation, and a sustained inflammatory milieu collectively drive a pathogenic cascade—from initial epithelial injury to immune activation and, crucially, the accumulation of matrix-producing myofibroblasts via processes like EMT and EndoMT. This ultimately leads to the exaggerated ECM deposition that defines the progression of pulmonary fibrosis in RA-ILD.

## 4 Treatment options and emerging therapies for RA-ILD

The optimal treatment strategies for RA-ILD remains under exploration, with immunomodulation and antifibrotic therapies being the primary approaches. Common treatments include corticosteroids, azathioprine, cyclophosphamide, mycophenolate mofetil, rituximab, and TNF-α inhibitors. Methotrexate, a first-line treatment for RA, is associated with concerns about acute pneumonitis, raising doubts about its role in RA-ILD development (53). Corticosteroids remain the cornerstone in RA-ILD management in clinical practice, working by modulating immune responses, reducing inflammatory cytokine production, altering lymphocyte distribution, and inhibiting macrophage activity (54). These effects are typically achieved through long-term oral administration or high-dose pulse therapy. Although clinical guidelines also recommended short-term high-dose corticosteroids as first-line therapy, this recommendation relies more on clinical experience than on solid evidence from comprehensive research (55).

The role of biologic disease-modifying antirheumatic drugs (bDMARDs) in ILD progression is still debated. While some bDMARDs, including biologics and targeted synthetic DMARDs (b/tsDMARDs), may exacerbate ILD due to immune responses and tissue damage, others offer therapeutic benefits by suppressing inflammatory cytokines. Early studies raised concerns about TNF-α inhibitors, such as infliximab, worsening ILD in RA patients (56), though later research did not support these findings. For example, a 2021 study found no significant link between conventional synthetic DMARDs (csDMARDs) or anti-TNF inhibitors and lung disease exacerbation. In contrast, non-TNF inhibitor bDMARDs like abatacept, rituximab, and tocilizumab have been shown to reduce the progression of lung disease in up to 90% of patients (57). The advent of Janus kinase inhibitors has provided new hope for RA-ILD management. Baker et al.’s study (58) indicated that tofacitinib reduced the incidence of ILD by fourfold, with a 69% lower risk of ILD compared to adalimumab. Moreover, a Japanese study highlighted the potential mechanisms by which biologics, including methotrexate and baricitinib, can delay ILD progression. In their vitro experiments revealed that this combination inhibited EMT and downregulated mesenchymal phenotype markers, providing a prominent direction for antifibrotic therapy (59).

In recent years, emerging immunotherapies—CD19 targeting chimeric antigen receptor (CAR)-T cell therapies—offer a serious opportunity to develop solutions for complex autoimmune diseases refractory to multiple immunosuppressant treatments, already had been used in the ILD associated with myositis (60) and diffuse systemic sclerosis (61). In André’s report (62), a pediatric patient with aggressive MDA5^+^ DM-RPILD achieved progressive long-term improvement and immunosuppressant-free remission over 11 months following the compassionate use of CD19 CAR-T cell therapy. Due to the potential of CD19-targeted CAR-T cell therapy in eliminating autoreactive B cells, disrupting pathological B-cell and T-cell responses, it holds promise for the early treatment of RA-ILD, potentially reversing outcomes.

Antifibrotic drugs delay the decline in lung function in patients with RA-ILD, and some of these drugs have been incorporated into clinical guidelines (63). Nintedanib, a tyrosine kinase inhibitor, has shown anti-fibrotic and anti-inflammatory effects in preclinical studies, slowing the progression of pulmonary fibrosis by inhibiting the release of pro-fibrotic, pro-inflammatory mediators, fibroblast migration and differentiation, as well as extracellular matrix accumulation (64). Eric et al. (65) found that nintedanib significantly slowed the decline in forced vital capacity (FVC) in RA-ILD patients with progressive fibrosis, and most adverse events, such as diarrhea, were manageable. Pirfenidone is an oral synthetic pyridine derivative known for its broad anti-fibrotic and anti-inflammatory properties. The TRAIL1 trial is the only multicenter, randomized, double-blind, placebo-controlled phase 2 clinical trial specifically designed to evaluate the safety, tolerability, and efficacy of pirfenidone in patients with RA-ILD. The study demonstrated Pirfenidon were even more significant in RA-ILD patients with UIP pattern, which even leds to a recommendation in the EULAR/ERS clinical practice guidelines 2025 for Pirfenidon in RA-ILD (63, 66). Additionally, inhibition of phosphodiesterase-4B (PDE4B) may be a novel approach for fibrosing ILDs such as RA-ILD (67). Nerandomilast is an orally administered preferential inhibitor of PDE4B with antifibrotic and immunomodulatory effects. It has been reported in a phase 2 trial involving patients with idiopathic pulmonary fibrosis, treatment with nerandomilast stabilized lung function over a period of 12 weeks (68).

## 5 Conclusions

This review summarizes the etiology of RA-ILD and its pathogenesis under different causative factors, enumerates the commonly used clinical drugs and recent pharmacological advances for the treatment at last. It holds significant implications for future directions. An in-depth understanding of their mechanistic interactions suggests that there should be a potential link between the pathogenesis of rheumatoid arthritis and ILD, involving multiple factors. Moreover, in the future treatment of RA, vigilance is warranted regarding the potential pulmonary progression associated with specific drugs. Additionally, the multiple shared pathogenic mechanisms of ILD and RA remind clinicians that while treating ILD, they must address the primary disease effectively, control the inflammatory levels in RA patients, and pay attention to the homeostasis of T/B cells. Finally, smoking cessation and reducing air pollution remain crucial measures for preventing the disease from an etiological perspective. By integrating these approaches, it is hoped that future treatment strategies for RA-ILD can achieve newer breakthroughs.
